# Back to the basics of ovarian aging: a population-based study on longitudinal anti-Müllerian hormone decline

**DOI:** 10.1186/s12916-016-0699-y

**Published:** 2016-10-03

**Authors:** A. C. de Kat, Y. T. van der Schouw, M. J. C. Eijkemans, G. C. Herber-Gast, J. A. Visser, W. M. M. Verschuren, F. J. M. Broekmans

**Affiliations:** 1 Department of Reproductive Medicine and Gynecology, University Medical Center Utrecht, Heidelberglaan 100, 3584 CX Utrecht, The Netherlands; 2 Julius Center for Health Sciences and Primary Care, University Medical Center Utrecht, Heidelberglaan 100, 3584 CX Utrecht, The Netherlands; 3 National Institute for Public Health and the Environment, Antonie van Leeuwenhoeklaan 9, 3721 MA Bilthoven, The Netherlands; 4 Department of Internal Medicine, Erasmus Medical Center, ‘s-Gravendijkwal 230, 3015 CE Rotterdam, The Netherlands

## Abstract

**Background:**

Anti-Müllerian hormone (AMH) is currently used as an ovarian reserve marker for individualized fertility counseling, but very little is known of individual AMH decline in women. This study assessed whether the decline trajectory of AMH is uniform for all women, and whether baseline age-specific AMH levels remain consistently high or low during this trajectory.

**Methods:**

A total of 3326 female participants from the population-based Doetinchem Cohort Study were followed with five visits over a 20-year period. Baseline age was 40 ± 10 years with a range of 20–59 years.

AMH was measured in 12,929 stored plasma samples using the picoAMH assay (AnshLabs). Decline trajectories of AMH were studied with both chronological age and reproductive age, i.e., time to menopause. Multivariable linear mixed effects models characterized the individual AMH decline trajectories.

**Results:**

The overall rate of AMH decline accelerated after 40 years of age. Mixed models with varying age-specific AMH levels and decline rates provided the significantly best fit to the data, indicating that the fall in AMH levels over time does not follow a fixed pattern for individual women. AMH levels remained consistent along individual trajectories of age, with an intraclass correlation coefficient (ICC) of 0.87. The ICC of 0.32 for AMH trajectories with time to menopause expressed the large variation in AMH levels at a given time before the menopause. The differences between low and high age-specific AMH levels remained distinguishable, but became increasingly smaller with increasing chronological and reproductive age.

**Conclusions:**

This is the first study to characterize individual AMH decline over a long time period and broad age range. The varying AMH decline rates do not support the premise of a uniform AMH decline trajectory. Although age-specific AMH levels remain consistently high or low with increasing age, the converging trajectories and variance of AMH levels at a given time before menopause shed doubt on the added value of AMH to represent individualized reproductive age.

**Electronic supplementary material:**

The online version of this article (doi:10.1186/s12916-016-0699-y) contains supplementary material, which is available to authorized users.

## Background

Women are born with an endowment of oocytes, which decreases as they age. The decline in oocyte quantity eventually leads to menopause, marking the end of the reproductive lifespan [1]. The ability to achieve spontaneous pregnancies ceases several years before the onset of menopause [2], and is thought to be related to the quality of remaining oocytes. With an ever-expanding societal tendency to delay childbearing to a later age, more women may thus unknowingly surpass their window of fertile years due to a decline in oocyte quality and quantity.

Over the past decades, many research efforts have aimed at quantifying the remaining pool of oocytes, otherwise known as the ovarian reserve. Anti-Müllerian hormone (AMH), produced by follicular granulosa cells, has recently emerged as a promising biomarker representing the number of remaining follicles in the ovaries [1]. Herein, AMH levels are suggested to provide an estimation of ‘reproductive age’, irrespective of chronological age [3–11]. In other words, a woman with a low AMH level would have a lower ovarian reserve, and thus a shorter time to menopause than a woman with a higher AMH level of the same (chronological) age. This concept has found its way into clinical practice, as women are currently receiving personalized family planning or fertility treatment counseling based on their AMH levels. Although this may seem like an advancement in reproductive healthcare, evidence to support this practice is lacking. Current knowledge of AMH is scarce, and limited by the use of a single measurement [12–18], small study populations [5, 8, 19], selected study groups rather than a population-based approach [5, 8, 12–18], theoretical rather than empiric models [20, 21], and restricted age ranges [12, 15]. The individualized use of AMH as an indicator of the reproductive lifespan is therefore still hampered by two main questions.

First, little is known about whether the rate by which AMH declines is the same for all women. In other words, are individual AMH decline trajectories parallel to one another, or not? Secondly, the value of a single AMH measurement remains elusive, can a woman with a high age-specific AMH level at 20 years be expected to also have a high age-specific value at age 35, and what does this mean for her trajectory with reproductive age, i.e., time to menopause? We aimed to answer these two questions by characterizing the longitudinal decline trajectories of AMH in relation to both chronological age and time to menopause in a large population-based study.

## Methods

### Study population

Our study population consisted of the female participants of the Doetinchem Cohort Study. The Doetinchem Cohort is a population-based cohort, whose participants were randomly recruited from the Doetinchem area of the Netherlands in 1987 [22]. The objective of the Doetinchem Cohort Study is to observe the impact of lifestyle and biological factors on chronic disease occurrence and quality of life [22]. At the time of recruitment, participants were aged between 20 and 59 years. After the baseline visit (round 1), participants were invited for follow-up every 5 years. At the time of the study, rounds 1 through 5 had been completed, leading to an approximate follow-up time of 20 years.

At each visit, lifestyle, general health, and reproductive history were assessed through extensive questionnaires, and biometric and laboratory measurements were performed. In addition to the laboratory measurements that were performed directly after each consecutive blood withdrawal, aliquots with additional plasma samples of each participant were immediately stored for future use. All participants provided written informed consent and ethical approval was granted by the Medical Ethics Committee of the Netherlands Organization of Applied Scientific Research. The use of stored sample specimens was ethically approved by the Ethical Committee for Biobank Studies of the University Medical Center Utrecht.

Only female participants from the Doetinchem Cohort with at least one available stored plasma sample, regardless of their age or menopausal status, were eligible for the current study. Of the total number of 4128 participating women, 3326 had an available plasma sample for at least one of the follow-up rounds. Rounds 1–5 comprised plasma samples of 3133, 2914, 2507, 2324, and 2051 women, respectively.

### AMH measurements

The plasma samples from round 1 were stored in EDTA aliquots at –30 °C. The samples derived from rounds 2–5 were stored in EDTA aliquots at –80 °C. Prior to the current study, the samples were thawed once for additional measurements and immediately refrozen. For the current study, stored plasma samples of rounds 1–5 were utilized. In March 2015, all the available samples of each participant had been retrieved from storage and were shipped on dry ice to AnshLabs (Webster, Texas, USA), where they were temporarily stored at –20 °C until the analyses were performed. AMH levels were measured with the picoAMH assay (AnshLabs), because of its low limit of detection and the small aliquot size necessary, which is crucial for cohort studies with a limited pool of biological samples. The plasma samples of each individual were measured in a single assay run, by a single laboratory operator. In total, two laboratory operators performed all measurements. At a mean level of 91.2 pg/mL, the coefficient of variation was 4.0 %. At 290.3 pg/mL, the coefficient of variation was 4.8 %. The limit of quantification was 3.0 pg/mL and the limit of detection 1.8 pg/mL. There were no indications of plate drift, with all coefficients of variation within plate columns and rows under 5 %.

### Time to menopause

Age at the time of the final menstrual period (FMP) was assessed by taking into account questionnaire information of cycle regularity, number of menstrual periods in the prior 12 months, oral contraceptive (OC) use, pregnancy, reproductive surgery, and self-reported age at menopause. Due to slightly differing questionnaires throughout the follow-up rounds, the assessment of the timing of the FMP differed per round. The earliest estimation of the timing of the FMP was considered to be the most accurate, being the most proximate to the event. Time to menopause was calculated by subtracting a participant’s age at the FMP from her age at follow-up. Women who ever underwent a bilateral oophorectomy were excluded from this calculation in order to obtain the time to natural menopause at each follow-up round. Women who underwent a hysterectomy before the onset of natural menopause were considered to have an unknown age at menopause.

### Missing data

For information on smoking, OC use, menstrual cycle regularity in rounds 1, 4, and 5, age at menarche, and body mass index (BMI), the percentage of missing information was below 2 %. In rounds 2 and 3, missing information for cycle status was 6.8 % and 14.6 %, due to missing information of the date of the last menstrual period. Missing information for hormone replacement therapy use increased with each round, and varied between 7.1 % and 59.8 %. Multiple imputation through predictive mean matching with 10 iterations was performed for these variables, including participant ID, age, and AMH levels solely as predictor variables, and all remaining variables both as predictors and outcomes. Multiple imputation was performed with R (http://www.R-project.org), using the ‘mice’ library (http://www.jstatsoft.org/v45/i03/).

### Assessment of individual decline rate: parallel or non-parallel trajectories

To assess whether the decline rate of AMH differed for individuals, AMH trajectories in relation to age and time to menopause were fitted with a mixed model approach using the ‘lme4’ package in R. Mixed models enable the evaluation of multilevel longitudinal data, and are thus able to take into account multiple measurements over time for each participant, with varying AMH levels (i.e., random intercept) and decline rates (i.e., random slope) for each individual. As AMH had a skewed distribution, AMH levels were logarithmically transformed. Levels below the detection limit of 0.0018 ng/mL were set at this level for the purpose of logarithmic transformation. _Log_AMH was used as the outcome of the mixed models, with chronological age or time to menopause as the time variable and participant ID as the group indicator variable. We modeled age and time to menopause with non-linear natural splines and checked the significance of non-linearity (a *P* value of < 0.05 indicated significant non-linearity). Models were adjusted for current OC use and OC use 5 years prior, hormone replacement therapy use, and current smoking and smoking 5 years prior, as these determinants were associated with the longitudinal AMH levels (data not shown). To decide whether women had differing age-specific AMH levels, differing decline rates, or both, the multivariable-adjusted models with a random intercept, slope or both, were compared to models with only fixed terms for these two parameters. The Akaike Information Criterion (AIC) of the models was used for this purpose. A lower AIC by at least 2 points represented a significantly better fit of the data.

### Assessment of the consistency of age-specific AMH levels: does high remain high and vice versa?

To get an indication of whether individual AMH levels that were relatively low or high based on age remained comparatively low or high as time progressed, women were divided into age-standardized AMH quartiles in round 1. The CG-LMS method, previously described in detail by Dólleman et al. [23], was used for age standardization. The AMH decline trajectory of women in these four quartile groups was then plotted against chronological age and time to menopause.

In order to measure whether the AMH levels of the individual participants remained on a single trajectory, and did not vary between the 95^th^ and 5^th^ percentile over time for example, the variance of AMH measurements within and between individuals was assessed for the final mixed models. By dividing the between-individual variance by the total variance (between-individual + within-individual variance), the intraclass correlation coefficient (ICC) was calculated. The ICC gives an indication of the correlation of AMH measurements on each individual’s trajectory, which is directly relative to the amount of variation between individuals. For AMH decline with age, we hypothesized that women would follow a consistent high or low trajectory, i.e., that the variance of their AMH levels around a trajectory would be low. We therefore postulated that most of the variance would arise from differences in AMH levels between individuals, in which case the ICC would approach 1. For AMH decline with time to menopause, we hypothesized that there would be little variance of AMH levels between individuals; for example, we expected that the AMH level at 10 years before menopause would be roughly similar across the whole group. In this case, the ICC would approach 0.

### Participant involvement

Participants of the Doetinchem Cohort were not directly involved in the formulation of the study question or realization of the study design. As this was a population-based study design, patients were not involved.

## Results

### Population characteristics

On average, women in the study population completed 3.1 visits, and 79 % of the participants completed two or more visits. The longest follow-up time was 21 years. In Table 1, the participant characteristics per follow-up round are presented. The number of women and their characteristics at each visit are listed per 5-year age groups in Additional file 1: Tables S1–S6. The youngest age at baseline was 20 years and the highest age at the end of follow-up was 81 years. At baseline, 18 % of the women were aged between 20 and 30 years, and 32 % were aged between 30 and 40 years. At each visit, the percentage of OC users and smokers decreased with increasing age (Table 1, Additional file 1: Tables S1–S3). The percentage of smokers within the same age categories (thus comprising different women at each visit) decreased over time. BMI levels increased both with age and over time within the same age categories, meaning that, on average, a 40-year-old woman had a lower BMI in 1987 than in 2007 (Table 1, Additional file 1: Table S4). Within the entire study population, the median (interquartile range (IQR)) ages at menarche and FMP were 13 (12–15) and 50 (48–53), respectively.

**Table 1:** Population characteristics per follow-up round

	Round 1 *n* = 3133	Round 2 *n* = 2914	Round 3 *n* = 2507	Round 4 *n* = 2324	Round 5 *n* = 2051
Age, years	40 ± 10	46 ± 10	50 ± 10	55 ± 10	59 ± 10
OC use	32	25	21	12	8
HRT use	0	4	4	2	2
Smoker	34	31	26	22	18
BMI, kg/m^2^	25 ± 4	26 ± 4	26 ± 4	27 ± 5	27 ± 5
Regular cycle^a^	60	54	45	20	10
Premenopausal	86	66	54	48	36
Pregnant	2	1	1	0	0

Numbers are given in % or mean ± SD

^a^OC users excluded

*OC* oral contraceptive, *HRT* hormone replacement therapy, *BMI* body mass index

By the end of follow-up (visit 5), there were 1882 women with a known age at menopause. In total, 440 women (13.2 %) underwent a hysterectomy, 139 women (4.2 %) had a unilateral oophorectomy, and 77 women (2.3 %) had a bilateral oophorectomy. The women with no available blood samples had similar baseline characteristics to the study population with regards to age, age at menarche, age at FMP, and OC use. They smoked more frequently at baseline (41.9 % vs. 33.6 %, *P* < 0.001) and had a higher average BMI (25.9 vs. 24.6 kg/m^2^, *P* < 0.001).

### General AMH decline

**Fig. 1:**
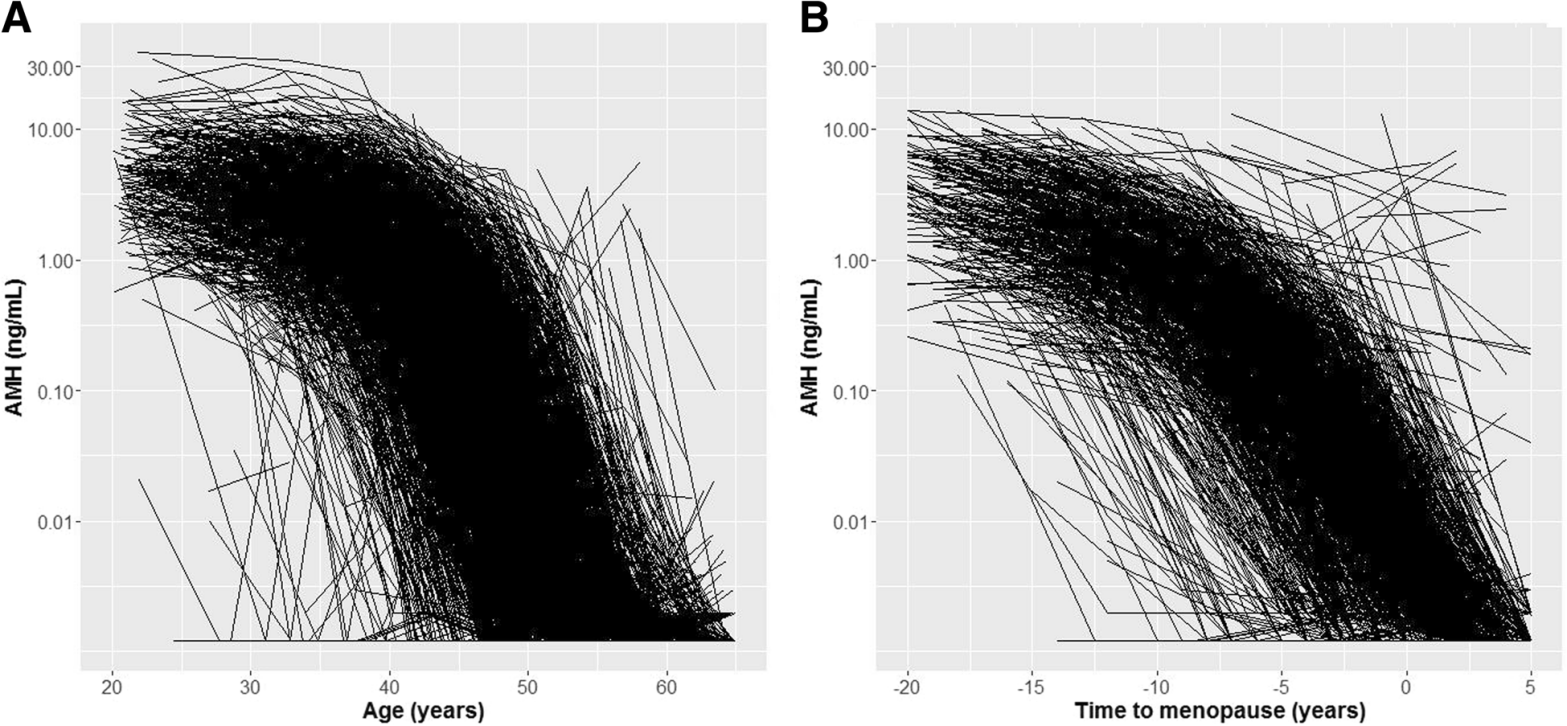
Decline of anti-Müllerian hormone (AMH) with chronological age (**a**) and time to menopause (**b**). Each line represents an individual trajectory based on observed AMH levels

AMH levels decreased with age and remained relatively stable over time within the visit-specific age categories (Additional file 1: Table S6). The percentage of AMH levels below the lower limit of detection reached a maximum of 88.3 % between the ages of 61 and 65 years (Additional file 1: Table S7). In Table 2, the observed AMH levels and number of measurements below the level of detection are listed for fixed chronological age and time to menopause points. Each individual AMH trajectory over age and time to menopause is depicted in Fig. 1. The decline patterns were not linear, but appeared to follow a sigmoid-shaped trajectory, indicating that the overall rate of AMH change differed depending on age and time to menopause. Generally, the decline rate appeared to be relatively low until 40 years, after which there was an apparent acceleration until a plateau at the lower limit of detection around the age of 55 was reached. There also appeared to be an acceleration of the overall decline rate with time to menopause at 10 years before the FMP, although this acceleration seemed more gradual than with age.

**Table 2:** Observed anti-Müllerian hormone (AMH) levels and proportion of undetectable levels at specified ages and time points before and after menopause

	AMH (ng/mL)	<0.0018 ng/mL
Age (years)		
20	3.86 (2.66–5.28)	0 (0)
30	2.84 (1.88–4.86)	0 (1)
40	0.93 (0.40–2.00)	1 (3)
50	0.01 (0.00–0.08)	28 (61)
60	0.00 (0.00–0.00)	82 (111)
Time to menopause (years)		
20	3.61 (2.25–6.28)	0 (0)
15	2.06 (0.94–3.28)	0 (0)
10	0.82 (0.50–1.51)	1 (1)
5	0.18 (0.08–0.34)	4 (7)
0	0.00 (0.00–0.01)	37 (76)
+5^a^	0.00 (0.00–0.00)	73 (139)

Numbers indicate median (IQR) and % (n), respectively

^a^5 years after the final menstrual period

### Assessment of individual decline rate: parallel or non-parallel trajectories

#### Chronological age

For AMH decline with age, the best fitting mixed models incorporated a random slope and random intercept and were significantly non-linear. The random slopes indicate that individual women had differing decline rates, and the non-linearity indicates that the overall rate of decline varied with age. Based on the multivariable-adjusted AMH levels, the rate of change in various age intervals was assessed per participant (Table 3). The average rate of AMH decline was greatest between 45 and 50 years of age.

**Table 3:** Estimated rate of anti-Müllerian hormone (AMH) change per time interval for age and per time interval to menopause, based on multivariable-adjusted mixed models

	5-year rate of change	1-year rate of change
Age period (years)		
20–25	0.05 ± 0.86 (–3.1 to 3.3)	0.01 ± 0.17 (–0.63 to 0.66)
25–30	–0.12 ± 0.78 (–2.9 to 2.8)	–0.02 ± 0.16 (–0.59 to 0.56)
30–35	–0.46 ± 0.60 (–2.6 to 1.9)	–0.09 ± 0.12 (–0.51 to 0.37)
35–40	–0.97 ± 0.35 (–2.0 to 0.42)	–0.19 ± 0.07 (–0.40 to 0.08)
40–45	–1.65 ± 0.13 (–2.3 to –0.3)	–0.33 ± 0.03 (–0.46 to –0.05)
45–50	–2.2 ± 0.37 (–3.5 to 0.3)	–0.43 ± 0.08 (–0.71 to 0.05)
50–55	–1.9 ± 0.46 (–3.6 to 0.3)	–0.38 ± 0.09 (–0.71 to 0.06)
55–60	–0.95 ± 0.37 (–2.4 to 0.4)	–0.19 ± 0.07 (–0.47 to 0.07)
Time to menopause period (years)		
20–15	–2.18 ± 0.65 (–3.75 to –0.30)	–0.44 ± 0.13 (–0.75 to –0.06)
15–10	–2.11 ± 0.48 (–3.25 to –0.70)	–0.42 ± 0.10 (–0.65 to –0.14)
10–5	–1.98 ± 0.17 (–2.58 to –1.39)	–0.39 ± 0.03 (–0.52 to –0.28)
5–0	–1.78 ± 0.32 (–2.87 to –0.80)	–0.36 ± 0.06 (–0.57 to –0.16)

Numbers indicate mean ± standard deviation (range) difference in individual _log_AMH levels between specified age and time to menopause intervals

**Fig. 2:**
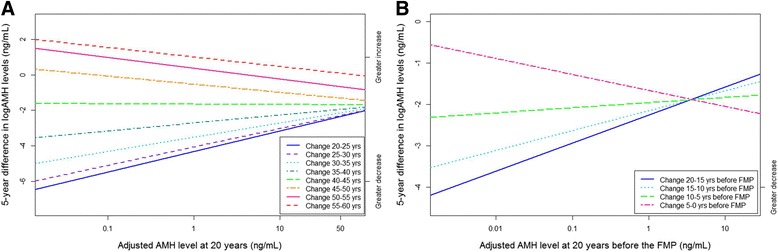
Association of multivariable-adjusted baseline anti-Müllerian hormone (AMH) level with rate of change in different time intervals. Colored lines indicate varying time intervals. **a** A higher AMH level at age 20 was associated with a slower decline rate between the ages of 20 and 25 (*blue line*), and a higher decline rate between the ages of 55 and 60 (*orange line*). **b** A higher AMH level at 20 years before the final menstrual period (FMP) was associated with a slower decline rate between 20 and 15 years before the FMP (*blue line*) and a higher decline rate in the 5 years before the FMP (*pink line*)

Because the AMH decline rates with age differed between participants, we plotted the adjusted AMH levels at age 20 against the decline rate at different age intervals in order to estimate whether there was a relationship between AMH levels and rate of decline. As seen in Fig. 2a, women who had a higher adjusted AMH level at age 20 had a slower decline rate between the ages of 20 and 25. This was also true between the ages of 25 and 40 years, whereas between the ages of 40 and 45 all women had approximately equal decline rates, regardless of their adjusted AMH levels at age 20. In contrast, after age 45, women with higher AMH levels at age 20 had a faster decline rate.

#### Time to menopause

For time to menopause, the best fitting mixed models incorporated a random slope and random intercept and were significantly non-linear. Thus, individual women had differing AMH levels and decline rates, and the rate of decline varied with time to menopause. In Table 3, the multivariable-adjusted rate of change per time to menopause interval is displayed, indicating that the average decline rate gradually decreased closer to the menopause. A higher AMH level at 20 years before the FMP was associated with a slower decline rate of AMH between 20 and 15 years before the FMP (Fig. 2b). In the last 5 years before the FMP, this relationship reversed, such that a high AMH level at 20 years before the FMP was associated with a faster AMH decline rate.

### Assessment of the consistency of age-specific AMH levels: does high remain high and vice versa?

#### Chronological age

**Fig. 3:**
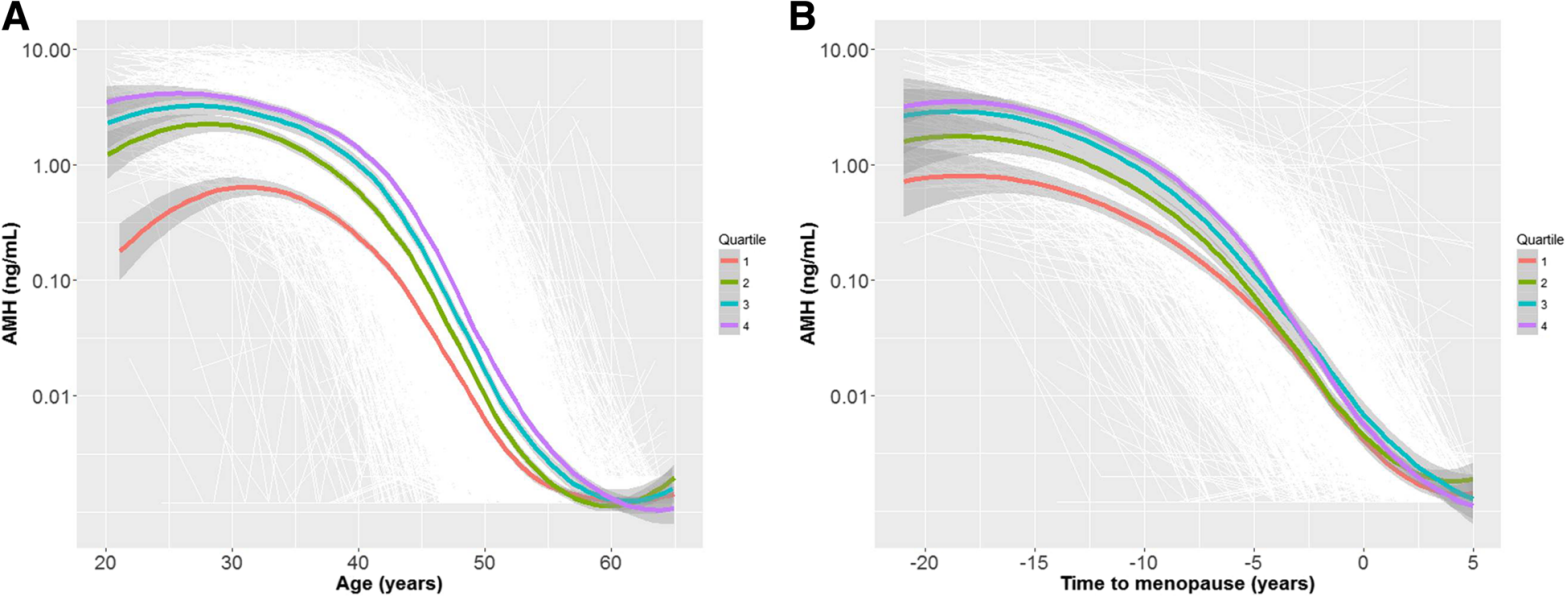
Anti-Müllerian hormone (AMH) decline with age and time to menopause. White lines represent individual trajectories based on observed AMH levels. Colored lines represent the group trajectories of women based on baseline age-specific AMH quartiles and gray areas indicate the standard error of a group trajectory. **a** The trajectories of women in the baseline age-specific AMH quartiles are distinguishable until they overlap between ages 55 and 60. The difference between women in low and high age-specific quartiles is largest at age 20. **b** The trajectories of women in the baseline age-specific AMH quartiles are distinguishable until they overlap around 5 years before the final menstrual period. The difference between women in low and high age-specific quartiles is largest at 20 years before the final menstrual period

According to the distribution of AMH with age at baseline, women were divided into age-specific AMH quartiles. The overall decline trajectory of each quartile group with chronological age is depicted in Fig. 3a. The difference between low and high age-specific AMH levels was maintained with increasing age, but the absolute difference became smaller. The ICC of the mixed model for AMH decline with age was 0.87, indicating that 13 % of the total variance could be accounted for by variability within individual AMH decline trajectories with age. Including only regularly cycling women at baseline (*n* = 2070), the ICC was 0.93, leaving 7 % of the total variance to be explained by variability within individual trajectories.

#### Time to menopause

The overall decline trajectory of each age-specific AMH quartile group with time to menopause is depicted in Fig. 3b. The difference between low and high age-specific AMH levels was distinguishable from 20 years before menopause to the FMP, but the absolute differences became smaller as women neared their FMP. The ICC for time to menopause was 0.32 for the whole group (*n* = 1882) and 0.31 for only the baseline regular cyclers (*n* = 1046), meaning that approximately one-third of the total variance arose from between-individual differences in AMH levels. Thus, differences between high and low age-specific AMH levels were also distinguishable before menopause, but there was more overlap of AMH trajectories with time to menopause than with chronological aging.

## Discussion

With this longitudinal study, we are able to shed light on the individual decline of AMH. We found that AMH trajectories with age and time to menopause were not identical, as the rate of decline differed between individuals. The rate of AMH decline was dependent on initial AMH levels, and this relationship differed with age and time to menopause. The evident differences between women with relatively high and low age-specific AMH levels at baseline became increasingly smaller as time progressed. The AMH levels of an individual woman correlated well with one another and thus did not deviate far from her trajectory. Contrary to our expectations, there was considerable variation of AMH levels between individuals with time to menopause. Taken together, these results indicate that women do not all follow the same decline trajectory of AMH, and that the largest differences in AMH levels between women can be found in early adult and reproductive life, after which they become increasingly smaller.

In a study of 81 fertile women, the variation of two AMH levels measured over an interval of 4 years was compared per study participant [24]. AMH levels were strongly associated with chronological age, and the change of individual AMH levels was comparable with the overall group decline (with a within-individual residual correlation of 0.66) [24]. Our results indicate that there is indeed a high within-individual correlation of AMH levels over a long trajectory with age, which appeals to the reliability of multiple AMH measures to estimate the trajectory of an individual.

Building an overall model for AMH decline with age previously proved to be a complex matter. In some cross-sectional studies, a quadratic decline function of AMH with age best fit the data [12, 14, 15, 18], while others built models with polynomials or flexible splines [6, 13, 16, 17]. Consequently, the proposed relationship of AMH with age took on various forms. Some models estimated the decline of age-specific AMH levels with age to be parallel [6, 14, 15], where others reported converging AMH levels with higher age [12, 13, 16–18]. With our longitudinal data, we show that the individual decline of AMH levels is more approximate to the latter observation, as the differences between low and high AMH levels decreased with age. The reality of the physiology of the decline of ovarian reserve furthermore appears more complex than could previously be studied, as the individual decline rates depended on both initial AMH levels and age or time to menopause.

The notion of differing decline rates of AMH was previously put forward by Faddy et al. [25], who suggested that the decline rate of follicles accelerates after reaching a certain numeric threshold. The number of follicles at this threshold was primarily estimated to be around 25,000 [26], and later hypothesized to differ per individual [25]. Thilagam [21] recently extended this mathematical model to include the influence of other hormones involved in ovarian aging. Indeed, our results indicate that there are more factors at play. We observed an average acceleration of AMH decline with chronological age, while the average decline rate gradually decreased with time to menopause. This could mean that follicle quantity changes are more dependent on chronological rather than reproductive aging, or could be an indication of increased AMH production per follicle due to increasing FSH levels nearer to menopause [27]. Moreover, higher initial AMH levels were first associated with a slower AMH decline rate, but later with a quicker decline. This could potentially mean that the suggested inhibitory effect of AMH on follicle recruitment [28–30] is effective up to a certain age or ovarian reserve threshold. This concept was previously brought up in a mouse study, in which the decline of growing follicles and AMH levels accelerated only later in reproductive life [31], leading to the hypothesis that compensatory mechanisms are present earlier on. In any case, this observation may prove detrimental for the hopes of improved reproductive age estimation with repeated AMH measurements.

Current time to menopause estimations with single AMH measurements are based on the concept that comparatively high (or low) AMH levels for age will remain high (or low) with age. Following this principle, a lower age-specific AMH level at any age should be associated with a shorter time to menopause [4, 6, 7, 9–11, 32]. While we did indeed observe differences between women in different baseline age-specific quartiles, it was surprising that there was such variability of AMH levels between women at a given time before menopause. This may in part explain the currently limited discriminatory capacity of AMH for time to menopause [32]. A related finding in this study is the 62.9 % observed AMH levels above the limit of detection within a year of the FMP. While this may partly be attributed to the high sensitivity of the assay, measurement error or recall bias for the timing of the FMP, earlier research has also suggested that the follicle pool is not entirely depleted at the time of menopause [2, 26, 33]. It may well be that this critical threshold differs between women, or that in the minority of the cases other causes such as hypothalamic dysregulation are at the root of the cessation of menses [2].

Prior longitudinal studies of AMH decline with age at menopause are in disagreement on whether AMH decline rate is associated with time to menopause [5, 8]. Contrary to our current findings, these studies assumed a linear decline of _log_AMH with time to menopause. The rate of decline was assessed over a maximum period of 14 years in 293 women [8], and over six annual intervals in 50 women [5]. A striking difference with our current study is the difference in age; Freeman et al. [8] included women aged between 35 and 48 (mean 41) years and Sowers et al. [5] included women with a mean age of 42 ± 2.7 years (no range provided). This age difference may explain the perceived linear decline, as we found the decline rate of AMH to be highest between the ages of 40 and 55, at which time the overall decline trajectory did appear more or less linear. Importantly, if AMH is ever to be used for individual estimations of the remaining number of fertile years in the light of family planning, the AMH measurements should occur at a far earlier age than in these studies. Our results indicate that the currently available prediction models with AMH decline rate cannot be extrapolated to the ages at which the measurement of AMH would be most useful. The added value of multiple AMH measurements for the prediction of time to menopause therefore requires further investigation.

This study is the first to characterize the longitudinal decline of AMH with regard to age and time to menopause, in the largest study population to date. Further strengths include the random selection of study participants, large age range, standardized data assessment, and storage of the samples from round 2 at –80 °C. There are no data on the effects of long-term storage on sample degeneration, but the selectivity of the picoAMH antibodies to a single binding site on the N-terminal domain of the AMH molecule minimizes the risk of measuring degradation products. The women with no available sample for AMH measurement smoked more often and had a higher BMI at baseline, suggesting that our results may represent a slightly more healthy part of the female general population. We were adequately able to correct for smoking status and BMI had no influence on AMH levels, and therefore do not believe this to have substantial consequences for the results presented here. As there were no women below the age of 20 in the cohort, with the majority of women above the age of 30 at baseline, our study is limited by the fact that we have relatively little information of ovarian reserve decline in the early years of female reproduction.

This longitudinal study reveals that there is no fixed decline trajectory of AMH. The decline rate of AMH differs between women and although age-specific AMH levels remain distinct, these differences decrease as age progresses. Moreover, the finding of varying AMH levels between women at the same time before menopause highlights the potential limitation of AMH for estimating time to menopause. Future studies of AMH as a predictor of the reproductive lifespan should consider the non-linear decline with age and time to menopause, the differences in rate of decline, and the converging, rather than parallel, trajectories.

In the light of reproductive aging and family planning, it would also be interesting to focus on the association of AMH decline and pregnancies. Levels of AMH were not associated with time to pregnancy in a prior study [34], and the wide range of AMH levels in proven fertile women [35] indicates that there is more to fertility than ovarian reserve. In a contemporary population-based cohort such as the Doetinchem Cohort Study, it is difficult to address this question due to the widespread use of contraceptives and the burden of collecting reliable information regarding the menstrual cycle, spontaneous pregnancies, infertility treatment, time to pregnancy, and age at last childbirth. Future studies, with a younger study population, may be better suited to answer this question.

## Conclusion

In conclusion, this study provides an insight into the physiology of ovarian reserve decline and paves the way for studies measuring the true feasibility of the individualized clinical use of single and multiple AMH measurements. Until then, AMH levels with regard to the reproductive lifespan should be interpreted with caution.

## Additional file

Additional file 1: Table S1. Number of women in each age category per follow-up round. Table S2. Current oral contraceptive users per age group and follow-up round (*n* (%)). Table S3. Current smokers per age group and follow-up round (*n* (%)). Table S4. Body mass index levels per age group and follow-up round (mean ± SD). Table S5. Current estrogen users for climacterial complaints per age group and follow-up round (*n* (%)). Table S6. Anti-Müllerian hormone (AMH) levels per age group and follow-up round in ng/mL (median [IQR]). Table S7. Number (%) of women with undetectable AMH levels (<1.8 pg/mL) per age category and follow-up round. (DOCX 22 kb)
