# Supplementary material for: Back to the basics of ovarian aging: a population-based study on longitudinal anti-Müllerian hormone decline
Source: BMC Med. 2016 Oct 3;14:151. doi: 10.1186/s12916-016-0699-y (PMC5046975; doi:10.1186/s12916-016-0699-y)
Supplement: Additional file 1: Table S1. — Number of women in each age category per follow-up round. Table S2. Current oral contraceptive users per age group and follow-up round (n (%)). Table S3. Current smokers per age group and follow-up round (n (%)). Table S4. Body mass index levels per age group and follow-up round (mean ± SD). Table S5. Current estrogen users for climacterial complaints per age group and follow-up round (n (%)). Table S6. Anti-Müllerian hormone (AMH) levels per age group and follow-up round in ng/mL (median [IQR]). Table S7. Number (%) of women with undetectable AMH levels (<1.8 pg/mL) per age category and follow-up round. (DOCX 22 kb) [file 12916_2016_699_MOESM1_ESM.docx]

**Additional file 1**

**Table S1.** Number of women in each age category per follow-up round

| **Age category** | **Round 1**  *n=3133* | **Round 2**  *n=2914* | **Round 3**  *n=2507* | **Round 4**  *n=2324* | **Round 5**  *n=2051* |
| --- | --- | --- | --- | --- | --- |
| 20-25 | 228 | - | - | - | - |
| 26-30 | 341 | 170 | - | - | - |
| 31-35 | 490 | 294 | 164 | - | - |
| 36-40 | 515 | 459 | 269 | 161 | - |
| 41-45 | 523 | 488 | 423 | 260 | 155 |
| 46-50 | 364 | 520 | 402 | 383 | 239 |
| 51-55 | 366 | 338 | 448 | 401 | 362 |
| 56-60 | 300 | 327 | 290 | 415 | 364 |
| 61-65 | - | 280 | 282 | 271 | 369 |
| 66-70 | - | 35 | 202 | 246 | 229 |
| 71-75 | - | - | 26 | 168 | 202 |
| 76-80 | - | - | - | 18 | 117 |
| 81-85 | - | - | - | - | 12 |

**Table S2.** Current OC users per age group and follow-up round (n (%)).

| **Age category** | **Round 1**  *n=3133* | **Round 2**  *n=2914* | **Round 3**  *n=2507* | **Round 4**  *n=2324* | **Round 5**  *n=2051* |
| --- | --- | --- | --- | --- | --- |
| 20-25 | 180 (89.1) | - | - | - | - |
| 26-30 | 194 (61.7) | 112 (70.9) | - | - | - |
| 31-35 | 175 (39.9) | 130 (47.3) | 81 (51.3) | - | - |
| 36-40 | 120 (25.7) | 144 (34.3) | 93 (36.9) | 104 (32.9) | - |
| 41-45 | 62 (13.9) | 116 (25.5) | 112 (28.4) | 67 (27.0) | 43 (28.7) |
| 46-50 | 34 (12.7) | 75 (16.4) | 91 (24.3) | 72 (20.7) | 49 (21.6) |
| 51-55 | 23 (10.1) | 19 (7.6) | 44 (11.5) | 40 (10.8) | 26 (7.9) |
| 56-60 | 1 (0.8) | 2 (1.0) | 2 (0.9) | 1 (0.3) | 4 (1.2) |
| 61-65 | - | 2 (1.5) | 2 (1.1) | 0 (0.0) | 0 (0.0) |
| 66-70 | - | 0 (0.0) | 2 (1.9) | 0 (0.0) | 0 (0.0) |
| 71-75 | - | - | 0 (0.0) | 0 (0.0) | 3 (2.7) |
| 76-80 | - | - | - | 0 (0.0) | 1 (1.9) |
| 81-85 | - | - | - | - | 0 (0.0) |

**Table S3.** Current smokers per age group and follow-up round (n (%)).

| **Age category** | **Round 1**  *n=3133* | **Round 2**  *n=2914* | **Round 3**  *n=2507* | **Round 4**  *n=2324* | **Round 5**  *n=2051* |
| --- | --- | --- | --- | --- | --- |
| 20-25 | 75 (72.8) | - | - | - | - |
| 26-30 | 142 (65.4) | 53 (31.2) | - | - | - |
| 31-35 | 196 (57.6) | 101 (34.4) | 49 (29.9) | - | - |
| 36-40 | 184 (52.7) | 148 (34.4) | 86 (32.0) | 36 (22.5) | - |
| 41-45 | 193 (57.1) | 175 (35.9) | 130 (30.7) | 71 (27.3) | 30 (19.4) |
| 46-50 | 103 (53.6) | 178 (34.2) | 124 (30.6) | 109 (28.5) | 58 (24.3) |
| 51-55 | 88 (53.7) | 97 (28.7) | 119 (26.6) | 104 (26.1) | 89 (24.7) |
| 56-60 | 69 (53.9) | 73 (22.3) | 63 (21.7) | 88 (21.3) | 66 (18.2) |
| 61-65 | - | 55 (19.6) | 51 (18.1) | 46 (17.0) | 54 (14.6) |
| 66-70 | - | 6 (17.1) | 33 (16.4) | 28 (11.7) | 28 (12.4) |
| 71-75 | - | - | 4 (15.4) | 21 (12.6) | 22 (10.9) |
| 76-80 | - | - | - | 6 (33.3) | 10 (8.8) |
| 81-85 | - | - | - | - | 2 (16.7) |

**Table S4.** BMI levels per age group and follow-up round (mean ± SD)

| **Age category** | **Round 1**  *n=3133* | **Round 2**  *n=2914* | **Round 3**  *n=2507* | **Round 4**  *n=2324* | **Round 5**  *n=2051* |
| --- | --- | --- | --- | --- | --- |
| 20-25 | 22.7 ± 3.2 | - | - | - | - |
| 26-30 | 23.2 ± 3.3 | 24.1 ± 4.3 | - | - | - |
| 31-35 | 23.6 ± 3.5 | 24.3 ± 3.8 | 25.2 ± 4.1 | - | - |
| 36-40 | 24.1 ± 3.5 | 24.6 ± 3.9 | 25.3 ± 4.1 | 26.1 ± 5.1 | - |
| 41-45 | 24.5 ± 3.4 | 25.1 ± 4.1 | 25.2 ± 4.0 | 25.7 ± 4.5 | 26.3 ± 5.0 |
| 46-50 | 25.5 ± 3.8 | 25.6 ± 3.7 | 25.9 ± 4.2 | 26.1 ± 4.3 | 26.3 ± 4.6 |
| 51-55 | 26.5 ± 4.1 | 26.4 ± 4.2 | 26.4 ± 4.0 | 26.3 ± 4.5 | 26.5 ± 4.5 |
| 56-60 | 26.7 ± 3.6 | 27.4 ± 4.4 | 26.8 ± 4.4 | 26.5 ± 4.2 | 26.5 ± 4.5 |
| 61-65 | - | 27.3 ± 4.0 | 27.6 ± 4.6 | 27.1 ± 4.5 | 26.7 ± 4.1 |
| 66-70 | - | 27.6 ± 4.2 | 28.0 ± 4.5 | 28.3 ± 5.2 | 27.7 ± 4.8 |
| 71-75 | - | - | 27.7 ± 3.9 | 27.8 ± 4.7 | 28.3 ± 5.1 |
| 76-80 | - | - | - | 28.3 ± 4.0 | 29.2 ± 5.9 |
| 81-85 | - | - | - | - | 29.2 ± 4.4 |

**Table S5.** Current estrogen users for climacterial complaints per age group and follow-up round (n (%)).

| **Age category** | **Round 1**  *n=3133* | **Round 2**  *n=2914* | **Round 3**  *n=2507* | **Round 4**  *n=2324* | **Round 5**  *n=2051* |
| --- | --- | --- | --- | --- | --- |
| 20-25 | - | - | - | - | - |
| 26-30 | - | 0 (0.0) | - | - | - |
| 31-35 | - | 1 (0.3) | 1 (0.6) | - | - |
| 36-40 | - | 2 (0.4) | 1 (0.4) | 2 (1.3) | - |
| 41-45 | - | 7 (1.4) | 2 (0.5) | 4 (1.6) | 1 (0.6) |
| 46-50 | - | 35 (6.9) | 12 (3.0) | 8 (2.1) | 8 (3.3) |
| 51-55 | - | 38 (11.8) | 50 (11.6) | 16 (4.0) | 7 (1.9) |
| 56-60 | - | 25 (7.8) | 23 (8.6) | 12 (2.9) | 8 (2.2) |
| 61-65 | - | 10 (3.6) | 11 (4.0) | 5 (1.9) | 2 (0.5) |
| 66-70 | - | 1 (2.9) | 3 (1.6) | 4 (1.6) | 3 (1.3) |
| 71-75 | - | - | 0 (0.0) | 3 (1.8) | 2 (1.0) |
| 76-80 | - | - | - | 0 (0.0) | 1 (0.8) |
| 81-85 | - | - | - | - | 0 (0.0) |

**Table S6.** AMH levels per age group and follow-up round in ng/mL (median [IQR]).

| **Age category** | **Round 1**  *n=3127* | **Round 2**  *n=2911* | **Round 3**  *n=2506* | **Round 4**  *n=2323* | **Round 5**  *n=2049* |
| --- | --- | --- | --- | --- | --- |
| 20-25 | 3.7 [2.2-5.8] | - | - | - | - |
| 26-30 | 3.6 [2.0-5.9] | 3.4 [1.8-5.5] | - | - | - |
| 31-35 | 2.8 [1.5-5.0] | 2.3 [1.2-4.0] | 2.2 [1.0-4.4] | - | - |
| 36-40 | 1.9 [0.9-3.8] | 1.6 [0.8-3.3] | 1.4 [0.7-2.7] | 1.4 [0.7-3.1] | - |
| 41-45 | 0.7 [0.3-1.7] | 0.6 [0.2-1.4] | 0.5 [0.2-1.4] | 0.5 [0.1-1.2] | 0.5 [0.1-1.5] |
| 46-50 | 0.1 [0.0-0.5] | 0.1 [0.0-0.3] | 0.0 [0.0-0.3] | 0.0 [0.0-0.3] | 0.0 [0.0-0.2] |
| 51-55 | 0.0 [0.0-0.0] | 0.0 [0.0-0.0] | 0.0 [0.0-0.0] | 0.0 [0.0-0.0] | 0.0 [0.0-0.0] |
| 56-60 | 0.0 [0.0-0.0] | 0.0 [0.0-0.0] | 0.0 [0.0-0.0] | 0.0 [0.0-0.0] | 0.0 [0.0-0.0] |
| 61-65 | - | 0.0 [0.0-0.0] | 0.0 [0.0-0.0] | 0.0 [0.0-0.0] | 0.0 [0.0-0.0] |
| 66-70 | - | 0.0 [0.0-0.0] | 0.0 [0.0-0.0] | 0.0 [0.0-0.0] | 0.0 [0.0-0.0] |
| 71-75 | - | - | 0.0 [0.0-0.0] | 0.0 [0.0-0.0] | 0.0 [0.0-0.0] |
| 76-80 | - | - | - | 0.0 [0.0-0.0] | 0.0 [0.0-0.0] |
| 81-85 | - | - | - | - | 0.0 [0.0-0.0] |

**Table S7.** Number (%) of women with undetectable AMH levels (<1.8 pg/mL) per age category and follow-up round.

| **Age category** | **Round 1**  *n=3127* | **Round 2**  *n=2911* | **Round 3**  *n=2506* | **Round 4**  *n=2323* | **Round 5**  *n=2049* |
| --- | --- | --- | --- | --- | --- |
| 20-25 | 1 (0.4) | - | - | - | - |
| 26-30 | 2 (0.6) | 1 (5.9) | - | - | - |
| 31-35 | 1 (0.2) | 4 (1.4) | 3 (1.8) | - | - |
| 36-40 | 6 (1.2) | 2 (0.4) | 3 (1.1) | 7 (4.3) | - |
| 41-45 | 11 (2.1) | 13 (2.7) | 9 (2.1) | 11 (4.2) | 13 (8.3) |
| 46-50 | 55 (15.1) | 89 (17.1) | 63 (15.7) | 75 (19.6) | 46 (19.2) |
| 51-55 | 198 (54.1) | 197 (58.3) | 262 (58.4) | 216 (53.9) | 202 (55.8) |
| 56-60 | 245 (81.7) | 274 (83.8) | 233 (80.3) | 331 (79.8) | 294 (80.8) |
| 61-65 | - | 240 (85.7) | 249 (88.3) | 221 (81.5) | 298 (80.8) |
| 66-70 | - | 11 (31.4) | 172 (85.1) | 204 (82.9) | 181 (79.0) |
| 71-75 | - | - | 5 (19.2) | 133 (79.2) | 170 (84.2) |
| 76-80 | - | - | - | 6 (33.3) | 96 (82.1) |
| 81-85 | - | - | - | - | 3 (25.0) |
